# SUBJECTIVE COGNITIVE DECLINE: FINE MOTOR DUAL-TASK OUTCOMES AND CHANGES IN CEREBRAL OXYGENATION

**DOI:** 10.1093/geroni/igae098.1056

**Published:** 2024-12-31

**Authors:** Talia Salzman, Kaya Kapilan, Diana Tobón, Sarah Fraser

**Affiliations:** University of Ottawa, Ottawa, Ontario, Canada; University of Ottawa, Ottawa, Ontario, Canada; Universidad de Antioquia, Medellín, Antioquia, Colombia; University of Ottawa, Ottawa, Ontario, Canada

## Abstract

Subjective cognitive decline (SCD) describes individuals who report cognitive complaints but perform normally on neuropsychological assessments. While the trajectory between SCD and objective cognitive impairments remains unclear, cognitive complaints are an important diagnostic criterion for mild cognitive impairment and dementia. An alternate method to measure cognitive function is dual-tasking. Dual-tasks, composed of a cognitive and motor component, may be more sensitive at detecting early cognitive declines and when paired with neuroimaging, capture changes in brain function. This study recruited older adults (70.0 ± 6.6 years) with SCD (n=24) and controls (n=18) who completed a working memory and finger tapping dual-task. In addition to completing a neuropsychological battery, finger tapping accuracy, cognitive response accuracy, and prefrontal cortex activity using functional near-infrared spectroscopy (fNIRS) were measured. Repeated measures ANOVAs were used to compare each variable across cognitive status (SCD, controls) and condition (single, dual-task). Findings revealed no differences in global cognition between the SCD and control group (p=.588). Despite this, greater prefrontal cortex activation was observed in the SCD group compared to the controls during the dual-task condition (p=.016). Cognitive and motor performance were worse in both groups as measured by lower tapping accuracy (p=.041) and response accuracy (p <.001) during the dual- compared to single task condition. Dual-tasks may be more sensitive than neuropsychological tests at detecting subtle changes in cognitive function between SCD and controls. Additional studies are needed to determine whether dual-task brain activity can complement existing measures of SCD to better identify older adults at risk of cognitive declines.

